# An analysis of bibliometric indicators to JCR according to Benford’s law

**DOI:** 10.1007/s11192-016-1908-3

**Published:** 2016-03-15

**Authors:** Alexandre Donizeti Alves, Horacio Hideki Yanasse, Nei Yoshihiro Soma

**Affiliations:** Aeronautics Institute of Technology – ITA, Praça Marechal Eduardo Gomes, 50 – Vila das Acácias, São José dos Campos, SP 12228-900 Brazil; Federal University of São Paulo – UNIFESP, Avenida Cesare Mansueto Giulio Lattes, 1201 – Eugênio de Mello, São José dos Campos, SP 12231-280 Brazil

**Keywords:** Journal Citation Reports, JCR, Bibliometric indicators, Benford’s law

## Abstract

Journal Citation Reports (JCR) is the main source of bibliometric indicators known by the scientific community. This paper presents the results of a study of the distributions of the first and second significant digits according to Benford’s law (BL) of the number of articles, citations, impact factors, half-life and immediacy index bibliometric indicators in journals indexed in the JCR Sciences and Social Sciences Editions from 2007 to 2014. We also performed the data analysis to country’s origin and by journal’s category, and we verified that the second digit has a better adherence to BL. The use of the second digit is important since it provides a more sound, complete and consistent analysis of the bibliometric indicators.

## Introduction

Bibliometric indicators have increasingly becoming of interest for they can be helpful in providing some measurement of the visibility of scientific publications. Bibliometric indicators can provide support in the evaluation of research success, the impact in the scientific community, and to research policy optimization. They can also help researchers in selecting the journals to which to submit their manuscripts (Durieux and Gevenois 2010). Funding agencies have also been looking at bibliometric indicators as they can offer quantitative measures to the results of the investment made in science (Cabezas-Clavijo et al. 2013).

As far as we know, the main source of bibliometric indicators known by the scientific community is the Journal Citation Reports (JCR). According to Thomson Reuters (2016), the owner of JCR, “Journal Citation Reports offers a systematic, objective means to critically evaluate the world’s leading journals, with quantifiable, statistical information based on citation data. By compiling articles’ cited references, JCR helps to measure research influence and impact at the journal and category levels, and shows the relationship between citing and cited journals”. JCR contains several bibliometric indicators, which can reveal information about the performance of each journal and annually it has the Science and Social Sciences editions.

The bibliometric indicators listed in the JCR have already been used in many studies in the last years (Vanclay 2012; Sangwal 2013; Campanario 2014, 2015). Given the interest in bibliometric indicators and the information that can be extracted from the JCR database, in this paper we investigate whether the main bibliometric indicators listed in the JCR comply with Benford’s law (BL).

BL is the empirical observation that in many data sets the significant digits are not uniformly distributed, as one might expect, but instead they tend to follow a very particular logarithmic distribution (Berger and Hill 2015). One of the applications of BL has been to aid researchers and professionals in identifying eventual anomalies in data sets, such as, financial data of religious community (Clippe and Ausloos 2012), the quality of occupational hygiene (De Vocht and Kromhout 2013), aggregated income taxes of Italian municipalities (Mir et al. 2014), birth time series (Ausloos et al. 2015), natural climatic process (Joannes-Boyau et al. 2015) and to distinguish different chaotic processes from stochastic processes (Li et al. 2015).

To the best of our knowledge, Campanario and Coslado (2011) was the first application of BL to bibliometric indicators listed in the JCR. They noted that the number of articles published, citations received to journals and impact factors of journals indexed in the JCR Science Edition from 1998 to 2007 not always comply with BL. They identified the first significant digit of each one of these indicators for each year separately, and compared them to the numbers predicted by BL.

In Alves et al. (2014), we extended the work of Campanario and Coslado (2011) analyzing the distribution of the first significant digit of the number of articles published of journals indexed in the JCR Science and Social Sciences Editions from 2007 to 2011. We also investigated their compliance with BL analyzing the number of articles published according to the country of origin and to the journal’s category.

Other studies that analyses bibliometric indicators using BL (first digit) are of Egghe and Guns (2012) and Hürlimann (2015). The former, introduced a generalization of BL based on the same data used by Campanario and Coslado (2011), while the latter suggests a truncated Erlang distribution and he partially used data given in Campanario and Coslado (2011) and Alves et al. (2014) to illustrate his new approach to some bibliometric indicators.

In this paper, we study the distributions of the first and second significant digits according to BL of several bibliometric indicators in journals indexed in the JCR Sciences and Social Sciences Editions from 2007 to 2014. The indicators considered are: number of articles, citations, impact factors, half-life and immediacy index. We also investigate their compliance with BL analyzing them indicators according to the country of origin and to the journal’s category.

## Benford’s law

BL, also known as the first digit law, is a logarithmic distribution function used to predict the first significant digit in numerical data. It asserts that the leading significant digit is not equally likely to be any one of the nine possible digits, but it is 1 more than 30 % of the time, and it is 9 less than 5 % of the time, with the probability of occurrence decreasing logarithmically in value as the digit increases from 1 to 9.

This was first observed by Newcomb (1881), who noted that the first pages of his book of logarithmic tables were more worn than the latter pages, which indicated that tables of logarithms were not used in a uniform way. However, only in 1938 the law was referred to as BL when Benford published a paper (Benford 1938) analyzing diverse data sets.

The general BL (Berger and Hill 2015) that specifies the probabilities of occurrence of the joint distribution of all the *m* significant digits is:

1 $$P\left( {D_{1} = d_{1} , D_{2} = d_{2} , \ldots , D_{m} = d_{m} } \right) = \log_{10} \left( {1 + \left( {\sum\limits_{j = 1}^{m} {d_{j} } 10^{m - j} } \right)^{ - 1} } \right) ,$$

for every positive integer *m*, where *d*_1_ is in $\{ 1,2, \ldots ,9\}$ and *d*_*j*_ is in $\left\{ {0,1, \ldots ,9} \right\}$ for all *j* ≥ 2.

Table 1 gives the expected proportions of BL for the first and second digits (generally referred to as the 1BL and 2BL), based on Eq. (1).

**Table 1:** Expected proportions of BL for the first and second digits

Digit (*d*)	*P* (*d*) First digit	*P* (*d*) Second digit
0	–	0.11968
1	0.30103	0.11389
2	0.17609	0.10882
3	0.12494	0.10433
4	0.09691	0.10031
5	0.07918	0.09668
6	0.06695	0.09337
7	0.05799	0.09035
8	0.05115	0.08757
9	0.04576	0.08500
Total	1	1

## Materials and methods

We get the data for this study from the JCR (Science and Social Sciences Editions) database available on the Web, covering the period from 2007 to 2014. Initially, we collected the following data for each journal: title, ISSN, year, edition, country of origin and the journal’s category. It is worth noting that a journal can belong to one, two, or more JCR categories. The following bibliometric indicators for all journals indexed in the JCR in both editions were also collected: number of articles, total of citations and self-citations, 2-year impact factor (IF) with self-citations (2Y-IF) and without self-citations (2Y-IF_WSC_), 5-year IF with self-citations (5Y-IF), cited and citing half-life, and immediacy index.

**Fig. 1:**
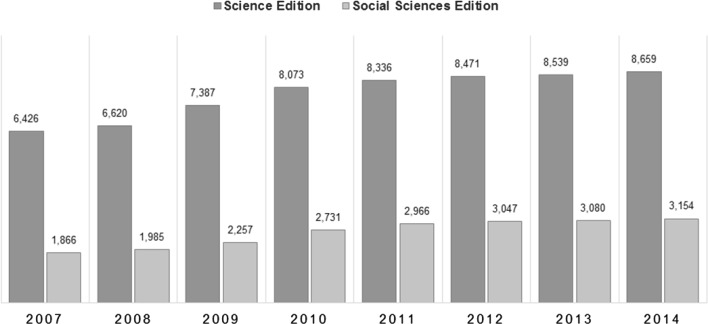
Total number of journals indexed in the JCR from 2007 to 2014

Figure 1 shows the total number of journals indexed in the JCR Science and Social Sciences Editions from 2007 to 2014. The actual number of journals considered in this study is smaller than the total available in the JCR, since we considered only those whose bibliometric indicators values are greater than zero. In addition, in the case of cited and citing half-life, all values equal to “>10.0” cannot be included in the study because it is not possible to obtain their significant digits directly. For these reasons, the number of journals considered varies for each bibliometric indicator. All impact factors where multiplied by 1000 and the half-life by 10 to avoid values smaller than 1.

We then identified the first and second significant digits of the bibliometric indicators of each journal indexed in the JCR, for each edition, to calculate their frequencies and, we compared them with the numbers predicted by BL. We also determined the Chi square test for 1BL and 2BL:

2 $$\begin{aligned} \chi^{2} \left( {n - 1} \right) & = \mathop \sum \limits_{i = k}^{n} \frac{{\left( {N_{\text{o}} \left( {d_{i} } \right) - N_{\text{e}} \left( {d_{i} } \right)} \right)^{2} }}{{N_{\text{e}} \left( {d_{i} } \right)}}, \\ & \quad k = 1 \,{\text{for}}\,1{\text{BL}}\,{\text{and}}\,k = 0 \,{\text{for}}\,2{\text{BL}}; \\ \end{aligned}$$

where $N_{\text{o}} \left( {d_{i} } \right)$ and $N_{\text{e}} \left( {d_{i} } \right)$ are the observed values from the data and expected values according to BL, respectively. For *n* = 9 we have *n* − 1 = 8 degrees of freedom, and *χ*^2^(8) = 15.507 for a 95 % confidence level. This is the critical value for the acceptance or rejection of the *Null Hypothesis*. For *n* = 10 we have *n* − 1 = 9 degrees of freedom, and *χ*^2^(9) = 16.919 for a 95 % confidence level.

We also tested each of the proportions separately using the *Z*-statistic, which verifies whether the observed proportion for a digit differs significantly from the expected value based on BL (Nigrini 2012). The *Z*-statistic has the following equation:

3 $$Z = \frac{{\left| {P_{\text{o}} - P_{\text{e}} } \right| - \left( {\frac{1}{2N}} \right)}}{{\sqrt {\frac{{P_{\text{e}} \left( {1 - P_{\text{e}} } \right)}}{N}} }} ,$$

where *P*_o_ denotes the observed proportion value, *P*_e_ the expected proportion value, and *N* is the total numbers of observations. The term (1/2*N*) is a continuity correction factor and it is considered only when it is smaller than the other term in the numerator. For a significant level of 5 %, the cutoff level is 1.96. The *Z*-statistic is important to recognize and indicate which numbers need further investigation.

## Results and discussion

**Fig. 2:**
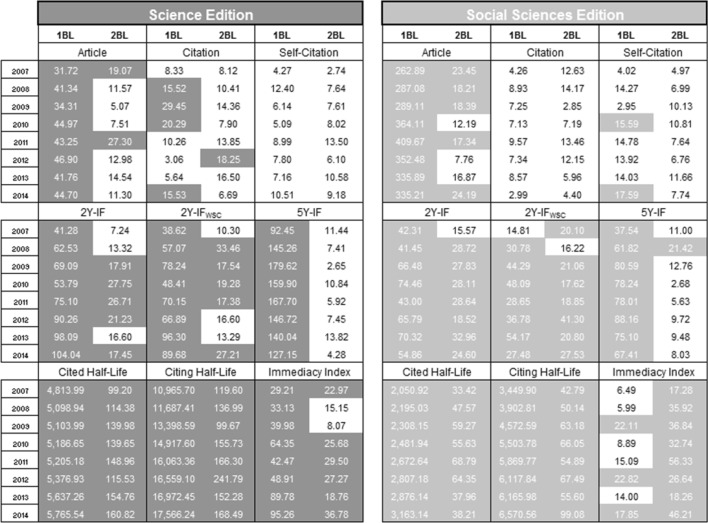
Chi square values for the 1BL and 2BL of the bibliometric indicators in journals indexed in the JCR from 2007 to 2014 (the *shaded numbers* indicate noncompliance with BL)

In Alves et al. (2014), we analyzed only the distribution of the first significant digit of the number of articles published of journals indexed in the JCR from 2007 to 2011. In this study, we extend our analysis to the second significant digit, and we investigate several other bibliometric indicators listed in the JCR from 2007 to 2014. In Fig. 2 we present a summary of our analysis. In the case of the number of articles, there is a small difference in relation to the values presented in Alves et al. (2014) because we considered now a larger number of decimal places in the calculation of the Chi square values. However, this difference did not affect the compliance (or not compliance) with BL in relation to our previous work.

According to all 288 tests performed, it is possible to observe that 191 (66.32 %) do not comply with BL. The result for each edition of the JCR is almost equal, 95 (65.97 %) for Science Edition and 96 (66.67 %) for Social Sciences Edition. By considering 1BL, 112 (77.78 %) do not comply with BL, while for 2BL 79 (54.86 %) do not comply with BL, which is a better result.

The average percentage of journals considered varied from an indicator to another. For instance, the total number of articles to the Science Edition is 97.82 % of the total number of journals for 1BL and 96.40 % for 2BL. For the Social Sciences Edition, the average percentage is very similar, 97.86 % for 1BL and 95.50 % for 2BL. The percentages are similar for the citations, self-citations, 2Y-IF, 2Y-IF_WSC_, 5Y-IF and immediacy index. However, for the cited and citing half-life the percentages are smaller than 77 % in the Science Edition and smaller than 63 % in the Social Sciences Edition.

### Number of articles

Campanario and Coslado (2011) noted that the number of articles published in journals indexed in the JCR Science Edition do not comply with 1BL from 1998 to 2007. Alves et al. (2014) also noted the same in journals indexed in the JCR Science and Social Sciences Editions from 2007 to 2011. The same occurred from 2007 to 2014, but for the 2BL, some Chi square values in some years are smaller than the critical value. For instance, only 2 years (2007 and 2011) do not comply in the JCR Science Edition.

The number of articles published in a journal varies a little from 1 year to another, since the number of issues in a journal seldom changes yearly. This may explain the non-compliance of the first digit and the slight improvement on the second digit for the number of articles published.

**Fig. 3:**
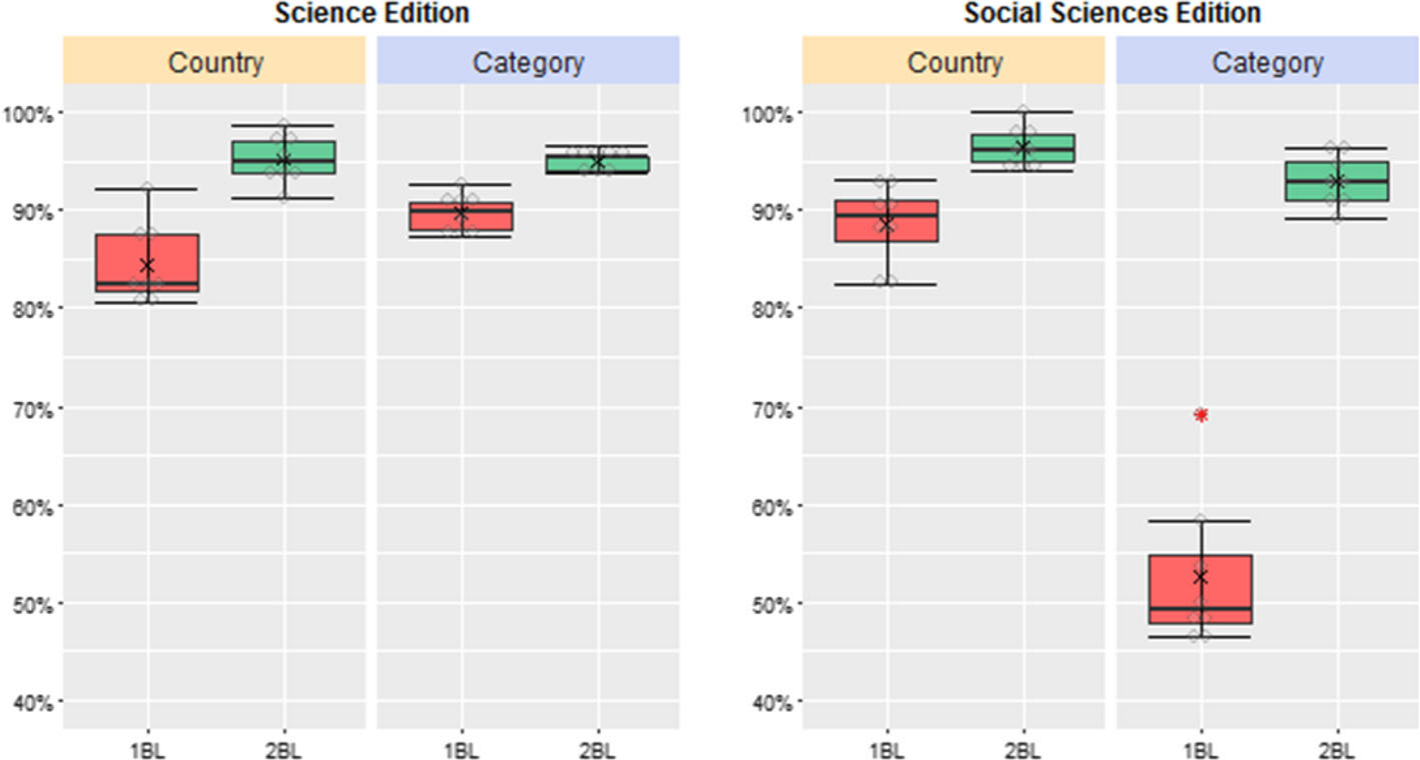
Percentage of countries and journal’s categories that complies with 1BL and 2BL for the number of articles published in journals indexed in the JCR

We also investigated the journals according to their country of origin and to their JCR category. In Fig. 3 we present the percentage of countries and journal’s categories that complies with 1BL and 2BL considering the Chi square values for the number of articles published in journals indexed in the JCR Science and Social Sciences Editions from 2007 to 2014. The results for the 1BL are generally very good, for 2BL the results are always better than 1BL. An outlier occurred for the 1BL in the journal’s category in the JCR Social Sciences Edition. In this case, the average percentage is 52.52 % for 1BL and 93.04 % for 2BL.

### Citation

The number of citations indicate the total number of times that each journal was cited by all journals included in the database within the current JCR year. For this indicator we observed that almost all data complies with 1BL, being the result still better for 2BL, that has a sole exception of non-compliance in the year 2012. Considering the self-citations, all years comply with 2BL. We also considered the number of citations without self-citations and to 2BL they all comply with BL. For the 1BL, non-compliance was observed in the years from 2009 to 2011 in the Science Edition. In 2009 and 2010, the Chi square values are smaller compared to those of the indicator with self-citations; nevertheless, they do not obey BL.

Considering the country of origin and the journal’s category for the citations and self-citations, both 1BL and for 2BL have a very high average acceptance percentage, around of 95 %, for the two JCR editions. For all cases, except by a small difference the journal’s category in Social Sciences Edition for self-citations, the percentage is higher for 2BL compared to 1BL.

### Impact factor

The IF identifies the frequency with which an average article from a journal receives citations by other articles in a particular period. To 2Y-IF, the last 2 years are considered and for the 5Y-IF the last 5 years. To the IF values we noticed that almost all of them do not follow 1BL, expect the year 2007 in the Social Sciences Edition for 2Y-IF_WSC_. Additionally, the 5Y-IF do not follow 1BL, but it follows the 2BL, except in 2008 in the Social Sciences Edition. This is interesting since neither 2Y-IF nor 2Y-IF_WSC_ follow BL in almost all years. It provides support for the preferential use of the 5Y-IF over the 2Y-IF or 2Y-IF_WSC_.

**Fig. 4:**
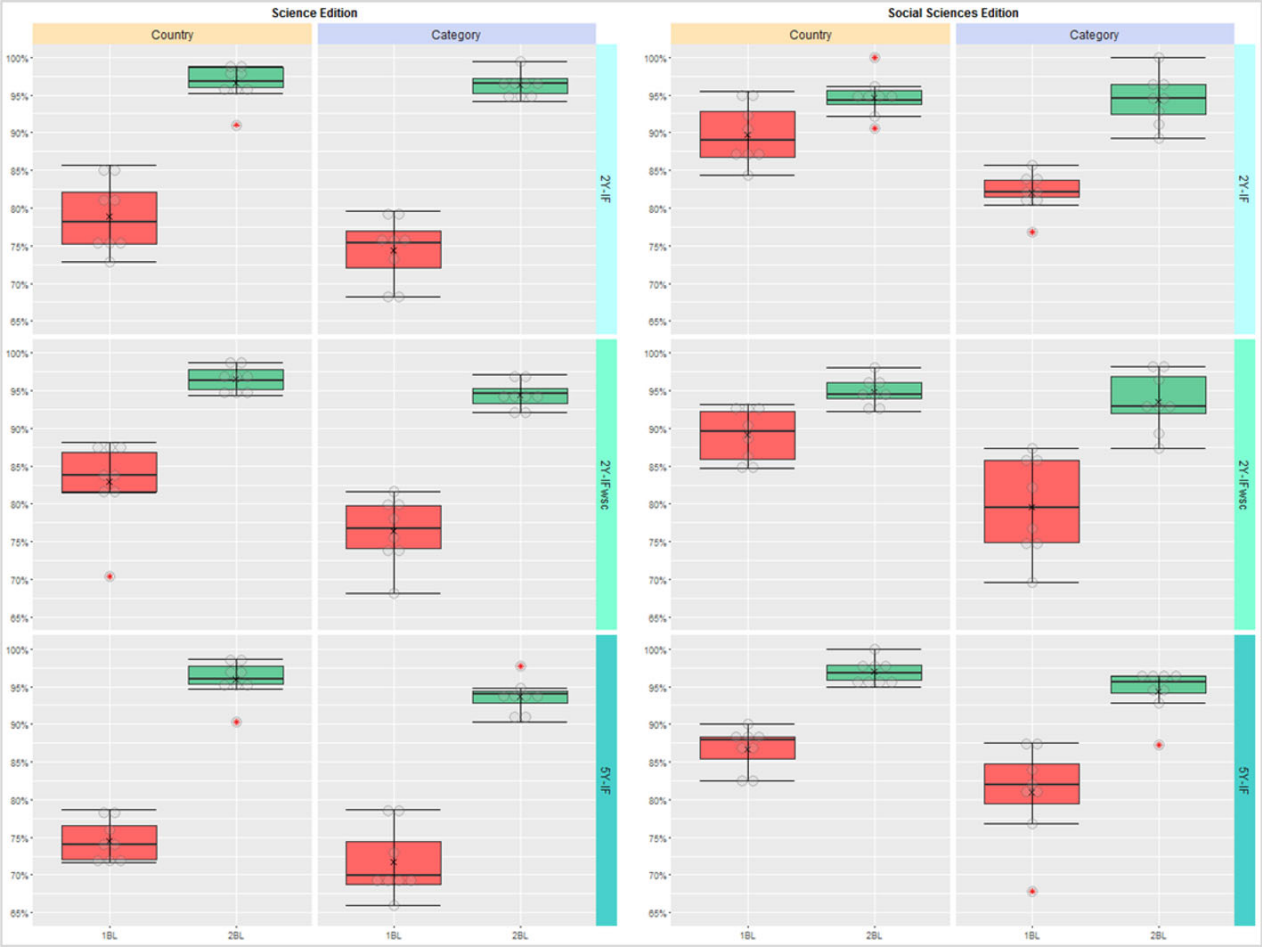
Percentage of countries and journal’s categories that complies with 1BL and 2BL for the 2Y-IF, 2Y-IF_WSC_ and 5Y-IF in journals indexed in the JCR

In Fig. 4 we present the percentage of countries and journal’s categories that complies with 1BL and 2BL considering the Chi square values for the impact factors in journals indexed in the JCR from 2007 to 2014. It is possible to observe that there is a significant difference from 2BL to 1BL in all cases, with an overall average percentage for compliance with 2BL around 95 %.

### Half-life and immediacy

The cited half-life gives the number of years back from the current year that accounts for 50 % of the total number of citations to a journal. The citing half-life identifies the number of years from the current year that accounts for 50 % of the cited references from articles published by a journal. The immediacy index measures how frequently, in average, an article from a journal is cited within the same year of publication and it is a useful metrics for evaluating journals that publish cutting-edge research.

We noted that not all years of the cited and citing half-life indicators comply with 1BL and 2BL for the two JCR editions. We expect that the great majority of half-life values “>10.0” is probably bounded from 10 to 20 years. For the sake of calculation we, therefore, assumed “1” to be the first digit of all the half-life values “>10.0”. The new Chi square values obtained are slightly smaller but the conclusions for the first digit remain the same.

For the immediacy indicator the same pattern occurred with 2BL, but to 1BL, the Chi square values are in accordance to BL for some years.

**Fig. 5:**
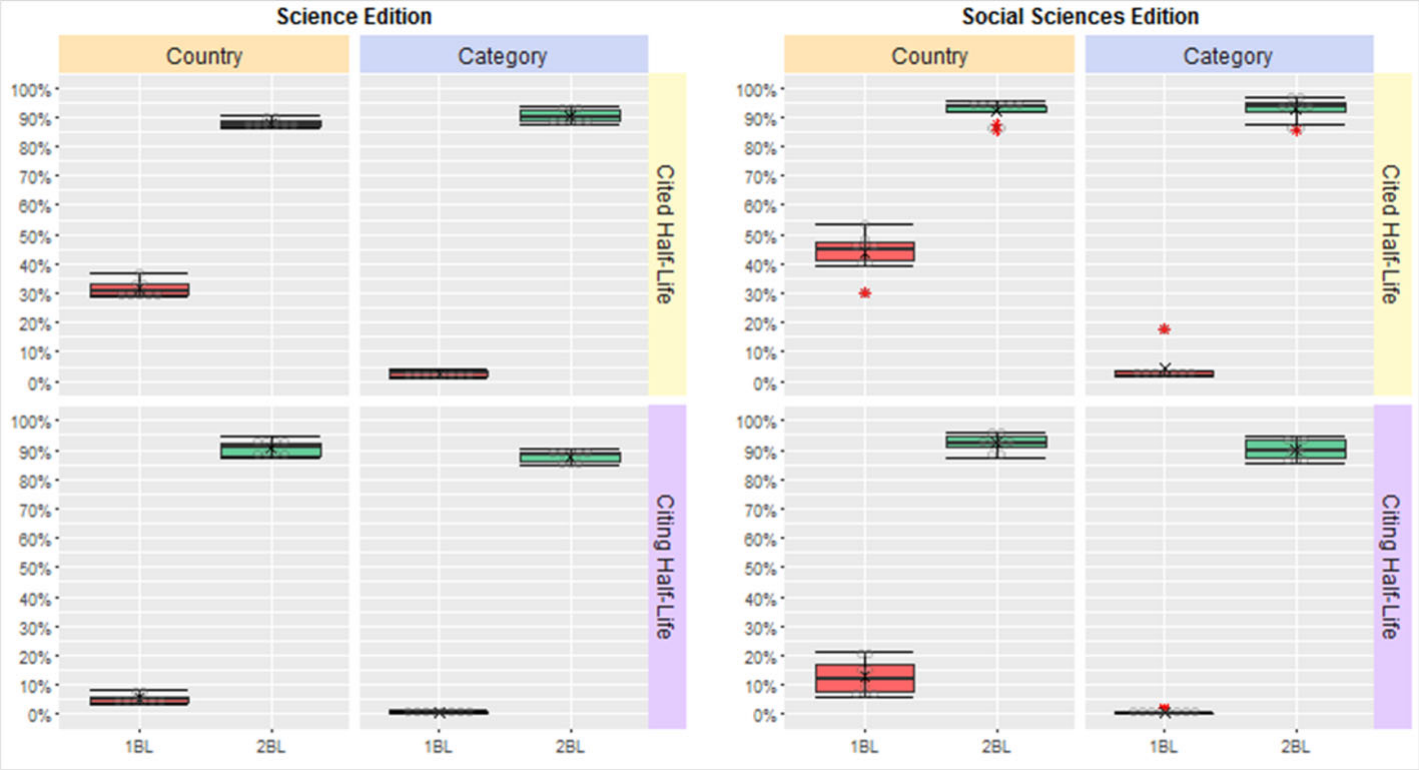
Percentage of countries and journal’s categories that complies with 1BL and 2BL for the cited half-life and citing half-life in journals indexed in the JCR

It is worth noting the differences in percentage of compliance of 1BL and 2BL of cited and citing half-life by country and journal’s categories shown in Fig. 5. We observed 1BL smaller than 1 % in some cases, while for the 2BL the percentage stays around 90 %. To the immediacy index, the average percentage is around of 90 % for the 1BL and 95 % for the 2BL, except in journal’s categories in the JCR Social Sciences Edition that the average percentages are the opposite, that is, around 95 % for 1BL and 90 % for the 2BL.

### *Z*-statistic

**Fig. 6:**
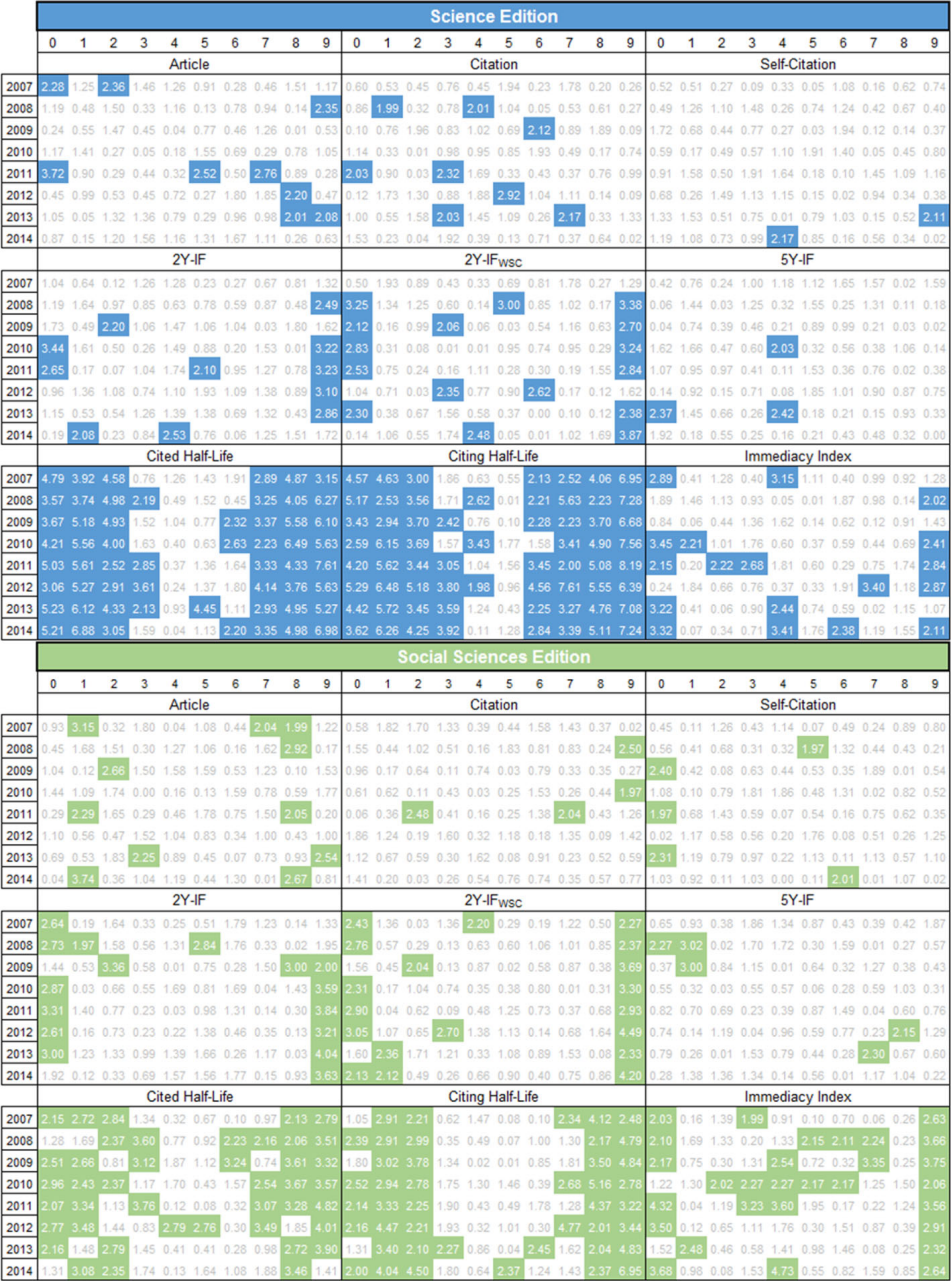
*Z* values considering the 2BL for the indicators in journals indexed in the JCR from 2007 to 2014 (the *shaded numbers* indicate values greater than the cutoff levels)

We used the *Z*-statistic to test each digit proportion separately, since it allows the identification of which ones may need further investigation. To 1BL, the result is that the *Z* values of almost all digits are greater than the cutoff level (1.96) for all bibliometric indicators but to citation and self-citations. Figure 6 presents the result to 2BL to all bibliometric indicators considered in this study. Observe that for digits 0 and 9, in almost all years, the values are greater than the cutoff value.

## Conclusions

In this paper, we analyzed the number of articles, citations, impact factors, half-life and immediacy index bibliometric indicators in journals indexed in the JCR Science and Social Sciences Editions from 2007 to 2014 according to 1BL and 2BL. We also performed the data analysis to country’s origin and by journal’s category.

In Alves et al. (2014) we verified that for countries and for journal’s categories the majority complies with 1BL. In this study, we verified that the second digit has a better adherence to BL, since the average percentage of compliance is around of 95 % to almost all bibliometric indicators. The non-compliance with 1BL of the number of articles for the period 1998–2007 was observed in Campanario and Coslado (2011), for the period 2007–2011 in Alves et al. (2014), and again in the current study for the period 2007–2014. Here we observed a slight improvement on the compliance with 2BL. This may be explained by the observation that the number of articles published in a journal varies a little from 1 year to another.

From the data analyzed an interesting result came out related to 5Y-IF. 5Y-IF has a better compliance to BL than 2Y-IF and 2Y-IF_WSC_. This result gives support for the preferential use of the 5Y-IF over 2Y-IF or 2Y-IF_WSC_. For both the cited and citing half-life they do not comply with 1BL and 2BL in all the years considered. The non-compliance with BL may be explained by the fact that we expect that these indicators to be quite stable over the years. The result for the immediacy index for some of the years was better for the 1BL compared with 2BL. This occurred also considering the journal’s category for the Social Sciences Edition. Finally, the result for citation and self-citations is very good, for both 1BL and for 2BL.

This study indicates that to consider the country of origin and journal’s category is relevant either to 1BL or to 2BL. The use of the second digit is important since it provides a more sound, complete and consistent analysis of the bibliometric indicators.

Further studies can be carried out using the generalized law of Benford (Egghe and Guns 2012).
